# Evaluating Prevalence and Patterns of Prescribing Medications for Depression for Patients With Obesity Using Large Primary Care Data (Canadian Primary Care Sentinel Surveillance Network)

**DOI:** 10.3389/fnut.2020.00024

**Published:** 2020-03-17

**Authors:** Svetlana Puzhko, Tibor Schuster, Tracie A. Barnett, Christel Renoux, Ellen Rosenberg, David Barber, Gillian Bartlett

**Affiliations:** ^1^Department of Family Medicine, Faculty of Medicine, McGill University, Montréal, QC, Canada; ^2^Department of Epidemiology and Biostatistics, INRS-Institut Armand-Frappier, Université du Québec à Montreal (UQAM), Laval, QC, Canada; ^3^Department of Neurology and Neurosurgery, McGill University, Montréal, QC, Canada; ^4^Department of Epidemiology and Biostatistics, McGill University, Montréal, QC, Canada; ^5^Centre for Clinical Epidemiology, Lady Davis Institute for Medical Research, Jewish General Hospital, Montréal, QC, Canada; ^6^Department of Family Medicine, Faculty of Medicine, Queen's University, Kingston, ON, Canada

**Keywords:** obesity, body mass index, depressive disorder, antidepressants, prescribing patterns, obesity bias

## Abstract

**Introduction:** Depression is a serious disorder that brings a tremendous health and economic burden. Many antidepressants (AD) have obesogenic effects, increasing the population of obese patients at increased risk for a more severe disease course and poor treatment response. In addition, obese patients with depression may not be receiving the recommended standard of care due to “obesity bias.” It is important to evaluate prescribing pharmacological treatment of depression in patients with obesity.

**Objectives:** To describe the prevalence and patterns of AD prescribing for patients with depression and comorbid obesity compared with normal weight patients, and to examine the association of prescribing prevalence with obesity class.

**Methods:** Study sample of adult patients (>18 years old) with depression was extracted from the national Canadian Primary Care Sentinel Surveillance Network (CPCSSN) Electronic Medical Records database for 2011–2016. Measures were prescribing of at least one AD (outcome) and body mass index (BMI) to categorize patients into weight categories (exposure). Data were analyzed cross-sectionally using descriptive statistics and mixed effects logistic regression model with clustering on CPCSSN networks and adjusting for age, sex, and the comorbidities.

**Results:** Of 120,381 patients with depression, 63,830 patients had complete data on studied variables (complete cases analysis). Compared with normal weight patients, obese patients were more likely to receive an AD prescription (adjusted Odds Ratio [aOR] = 1.17; 95% Confidence Interval [CI]: 1.12–1.22). Patients with obesity classes II and III were 8% (95% CI: 1.00, 1.16) and 6% (95% CI: 0.98, 1.16) more likely, respectively, to receive AD. After imputing missing data using Multiple Imputations by Chained Equations, the results remained unchanged. The prevalence of prescribing >3 AD types was higher in obese category (7.27%, [95% CI: 6.84, 7.73]) than in normal weight category (5.6%; [95% CI: 5.24, 5.99]).

**Conclusion:** The association between obesity and high prevalence of AD prescribing and prescribing high number of different AD to obese patients, consistent across geographical regions, raises a public health concern. Study results warrant qualitative studies to explore reasons behind the difference in prescribing, and quantitative longitudinal studies evaluating the association of AD prescribing patterns for obese patients with health outcomes.

## Introduction

Depression is a serious medical disorder that brings a tremendous health and economic burden to society. The considerable health burden of depression includes significant morbidity, reduced functioning, poor quality of life and increased mortality, especially from suicide (1). Moderate and severe depression has been associated with 50–75% higher per capita costs of health care (2). The overall prevalence of lifetime depression in Canada was estimated at 11.3% in 2012 (3). The prevalence of treatment resistant depression in Canadian primary care is 21% (4); moreover, the individual response to treatment for certain antidepressants (AD) is unknown. One of the clinical markers for ineffective AD treatment may be patient's weight. In Canadian primary care, treatment resistant depression is overrepresented by obese and overweight patients (4). Several studies reported that obese patients responded poorly to AD medications, with some studies reporting different response to individual AD types in obese patients, especially those with morbid obesity (4–6), compared with normal weight patients. This potential difference in response is, however, not yet reflected in the guidelines (7–10). On the other hand, many AD have obesogenic effects, increasing the population of obese patients at elevated risk for poor response to treatment (11–13). This negative cycle contributes both to the prevalence of treatment resistant depression and the obesity epidemic.

In Canada, nearly 60% of adults are overweight and almost one-quarter (23%) are obese (14, 15). Both obesity and depression are among the leading causes of preventable diseases and disability worldwide. Obese patients with depression constitute a highly stigmatized population with low self-esteem, poor quality of life, frequent use of health services, and decreased involvement in the labor force (2, 16, 17). Even though several studies suggest that obese patients respond to AD differently compared with normal weight patients and, therefore, may need special approach to treatment, there are no current guidelines on treatment of depression tailored to obese patients, except for those with eating disorders. In addition, the population of obese patients may face an important problem in receiving an adequate standard of medical care due to a phenomenon labeled the “obesity bias” which originates from unsubstantiated beliefs that obese and overweight patients are irresponsible and less likely to be adherent to treatment (18, 19). Hence, treatment of obese patients with comorbid mental conditions may be suboptimal and may negatively affect their health outcomes (20). It is imperative, therefore, to evaluate the prevalence and patterns of prescribing pharmacological treatment to patients with depression and comorbid obesity and to examine the association between obesity and AD prescribing. To our knowledge, very few studies (21, 22) evaluated how health providers prescribe AD to obese and overweight patients with depression; they showed that utilization of AD may be contributing to population-level increases in excess weight [(22), UK] and that obese patients are less likely to receive recommended standards of care [(21), USA]. To our knowledge, no study evaluated the prevalence and patterns of AD prescribing to patients with obesity in Canada or the association of certain prescribing patterns with the class of obesity. Depression is most commonly diagnosed, managed and treated in primary care in Canada (23). Primary care is usually an entry point to depression treatment, due to ease of access to a primary care providers (PCP) (compared with access to a specialist), lack of specialists in a patient's residential area, or long waiting time to see a specialist (4, 24). Prescribing AD is a common practice for many primary care providers (25), and most of AD prescriptions in Canada are issued by PCP (23).

The goal of the present study is to describe the prevalence and patterns (number of AD types prescribed) of AD prescribing for Canadian primary care patients diagnosed with depression who have comorbid obesity compared with normal weight patients with depression, and to examine the association of prescribing prevalence with obesity status, including obesity class. Study results are expected to generate hypotheses for further longitudinal studies evaluating the association of patterns of AD prescribing for obese patients in Canada with health outcomes. The focus will be on AD known for their risk to increase weight and AD shown to have different treatment outcomes in obese patients.

## Methods

### Data Source and Study Population

For this study, we used Canadian Primary Care Sentinel Surveillance Network **(**CPCSSN), a large pan-Canadian primary care database that combines de-identified patients' electronic medical records (EMRs) data from 12 primary care practice-based research networks across Canada, spanning 8 provinces and 1 territory (15, 26, 27). CPCSSN extracts primary care data on a regular (quarterly) basis from different EMR products and transforms it into a common database in a central source (26, 27). By May 2016, nearly 1200 sentinels from over 200 practice sites participated in CPCSSN; the database included demographics, encounter diagnoses, lab results, referrals, procedures, and prescriptions for more than 1.5 million patients (15). To address problems that may arise from EMR-data related issues, such as unstandardized data entry and free-text documentation, CPCSSN applies extensive cleaning algorithms (26).

Although a substantial part of data on patients' body mass index (BMI) is missing in CPCSSN, this database contains more BMI records than the objective BMI measurements collected by Statistics Canada health surveys over the past twenty years (15). CPCSSN is considered to be representative of the general Canadian population, albeit older adults are over-represented and young adult males are under-represented (27).

The population of adult patients with life-time depression was extracted from the CPCSSN database for the period June 2011–June 2016. All adult patients (patients who were 18 years of age or older as of June 2011) with depression who had at least one encounter with their primary care provider (PCP) within this period were included. To select patients with lifetime depression, a CPCSSN definition of depression and a validated case detection algorithm (28) were applied. The algorithm combines information from patients' problem list [Encounter Diagnosis Codes, used by some providers/sites to record the information on diagnosis (29)], prescription records, and billing [Billing Diagnosis Codes, used by other providers/sites to record the information on diagnosis (29)]. This algorithm detects lifetime depression, including an ongoing depression episode or a history of depression (28). CPCSSN case definition for depression was shown to have a sensitivity of 81.1 (95% CI: 77.2–85.0) and a specificity of 94.8 (95% CI: 93.7–95.9) (28).

### Measures

#### BMI and Weight Category

BMI was calculated in CPCSSN as body weight in kilograms divided by the square of the height in meters. We used the first record of BMI in CPCSSN for the study period to minimize possible misclassification of exposure (weight groups) due to weight-modulating effects of certain AD. BMI was used as a continuous exposure variable and was categorized into weight categories using WHO and Health Canada standards: 25–29.99 kg/m^2^ = overweight, ≥30 kg/m^2^ = obese, 18.5–24.99 kg/m^2^ = normal, <18.5kg/m^2^ = underweight. Extreme outliers (70 kg/m^2^ < BMI < 15 kg/m^2^) representing values outside plausible ranges were excluded. In addition, patients with obesity were subdivided into three classes. Class I comprises patients with BMI of 30–34.99 kg/m^2^, class II contains patients with BMI values between 35 and 39.99 kg/m^2^, and class III includes patients with BMI equal or greater than 40 kg/m^2^.

#### Socio-Demographic and Health Data

Patients' age (continuous variable and categorized into 6 age groups: 18–25 years, 26–35 years, 36–45 years, 46–55 years, 56–65 years, and >65 years of life), sex (dichotomous variable, men/women), and postal code (proxy for rural or urban settings) were applied to characterize patients by weight category. Following Canada Post's procedure for classification, residence in rural or urban areas was determined using the second digit of the first 3 digits practice's postal code (so-called forward sortation areas) assigning “rural” to those who had a value of zero and urban to those with other values. Network identification number (ID) was used to stratify patients attending practices belonging to different networks. The comorbidities measured at baseline included health conditions for which validated case definitions were developed by the CPCSSN: dementia, diabetes, osteoarthritis, hypertension, chronic obstructive pulmonary disease (COPD), Parkinson's disease, and epilepsy. The variable “comorbidities” was further categorized into two categories: (1) no comorbidities; (2) at least one comorbidity. The life-style variable “smoking status” had 66% of missing data and, therefore, was not retained for complete case (CC) analysis. The missing data for this variable were subsequently imputed, and the analyses were repeated for the whole sample of patients with depression, with and without adjustment for smoking status.

#### Antidepressant Prescription

Medications in the CPCSSN database are assigned World Health Organization (WHO) Anatomical Therapeutic Chemical (ATC) codes. Respectively, AD are assigned ATC NO6A code (30). The first record of prescription of any of AD recommended by the most recent (2016) Canadian Network for Mood and Anxiety Treatments (CANMAT) guidelines (10) (Supplementary Table 1) during the study period was included in the analysis. There was no washout period for AD use, and our sample was a sample of prevalent users, including both current and new users of AD.

### Statistical Analysis

#### Sample Description, Overall and by Weight Categories

To characterize participants with lifetime depression within each of the four weight categories and to compare their baseline socio-demographic and health characteristics, descriptive statistics were reported. Categorical variables were described using frequencies and percentages. Continuous variables were described using means and standard deviations or medians and interquartile ranges, as appropriate. As the primary purpose was data description, exploration and generation hypothesis, no confirmatory hypothesis tests were conducted. Focus was given on descriptive analysis, with an emphasis on the clinical importance of absolute differences and 95% confidence intervals (95% CI).

#### Evaluating Prevalence of AD Prescribing for Patients Belonging to Different Weight Categories

Period prevalence of AD prescribing was calculated for patients with lifetime depression belonging to different weight categories for the 2011–2016 period. The denominator was the number of patients in each of the four weight categories with lifetime depression extracted from the CPCSSN database. The numerator comprised patients of the same weight category who were prescribed AD. The presence or absence of exposure to AD was established by evaluating if there was at least one prescription for any of the relevant AD (Supplementary Table 1) in 2011–2016. A subgroup analysis was performed for obese patients (BMI > 30 kg/m^2^) according to the degree of obesity (classes I, II and III). Stratification by age groups, sex, and presence of at least one comorbidity was applied. Differences in frequency distributions and proportions were described numerically, including 95% CI, and were illustrated graphically.

#### Association of Obesity Status With Prevalence of AD Prescribing by Regression Analysis

The association between the obesity status and the prevalence of AD prescribing was examined in a multivariable logistic regression adjusting for age, sex, and comorbidities. The exposure variable “weight” was created with 4 categories: underweight, normal weight, overweight, and obese, with normal weight as a reference category. The outcome was prescribing at least one AD (yes/no). Age, sex, and comorbidities were included as a priori important clinical variables and were retained in the final model. Two types of regression models were applied: (1) logistic regression, without adjustment for network ID, to estimate a marginal national trend in prescribing; (2) mixed effects logistic regression model with random intercept and fixed effects, adjusting for clustering within networks.

#### Subgroup Analysis for Patients From Different Networks

Since different networks belong to different Canadian provinces that may have substantial differences in drug coverage and other factors, we performed a subgroup analysis to evaluate whether there is a consistency of the association between obesity status and AD prescribing prevalence between networks. To ensure consistency of data between network ID and Residence Postal Code, subgroup analysis was conducted for patients without missing data on Residence Postal Code variable (*n* = 62,020).

#### Imputing Missing Data for Weight and Smoking Status

To evaluate the possible impact of missingness of data on weight and smoking status on the effect estimates, we applied multiple imputation by chain equations (MICE) to the total sample of patients with depression, using the “mice” package for the statistical program “R” version 3.5.2 (31). The number of imputed datasets was 5, and the Predictive Mean Matching (“pmm”) method was applied to impute missing data for weight and smoking status. The following variables were used in the imputation model: age, sex, comorbidities, network ID. The five imputed datasets were then used to build the regression models for the associations between weight status and AD prescribing, and the obesity classes and AD prescribing. The results were then pooled, and the pooled effect estimates and 95% CI were reported and compared with the CC analysis.

## Results

Data from 120,381 people with life-time depression who had an encounter with their PCP between June 2011 and June 2016 were extracted from the CPCSSN database.

### Population Characteristics

Of 120,381 patients with depression, 63,830 patients had complete data on BMI, sex, age, comorbidities, and prescribed medications and were included in the CC analysis. Their characteristics are shown in Table 1. Among the patients excluded from the CC analysis, 46.8% (56,387 patients) lacked the data on weight, 0.02% (29 patients) on sex, 64.2% (77,296 patients) on smoking, and 3.4% (4,087 patients) on postal codes.

**Table 1:** Characteristics of patients with depression belonging to different weight categories.

	**Weight category**				**Total** ***N* = 63,830** **n (%)**
	**Underweight** ***N* = 1,685 (2.6%)** **n (%)**	**Normal weight** ***N* = 23,188 (36.3%)** **n (%)**	**Overweight** ***N* = 19,643 (30.8%)** **n (%)**	**Obese** ***N* = 19,314 (30.5%)** **n (%)**	
**Age**					
mean (SD)	32.9 (17.2)	38.3 (15.9)	43.3 (15.5)	42.1 (14.5)	40.9 (15.6)
median (IQR)	25.4 (22.1)	35.3 (24.4)	42.4 (22.9)	40.9 (21.0)	39.3 (23.5)
**Sex**					
men	380 (22.6%)	5,569 (24.0%)	6,982 (35.5%)	5,791 (30.0%)	18,722 (29.3%)
women	1,305 (77.5%)	17,619 (76.0%)	12,661 (64.5%)	13,523 (70.0%)	45,108 (70.7%)
**BMI, first measure**					
mean (SD)	17.5 (0.8)	22.2 (1.7)	27.3 (1.4)	36.1 (6.2)	27.8 (6.9)
median (IQR)	17.7 (1.1)	22.4 (2.8)	27.2 (2.4)	34.2 (6.6)	26.5 (8.1)
**Comorbidities**					
At least one comorbidity	126 (7.5%)	1,884 (8.1%)	2,451 (12.6%)	3,891 (20.2%)	8,352 (13.1%)
COPD	38 (2.3%)	339 (1.5%)	302 (1.5%)	457 (2.4%)	1,136 (1.8%)
Dementia	19 (1.1%)	199 (0.9%)	235 (1.2%)	212 (1.1%)	665 (1.0%)
Diabetes	14 (0.8%)	314 (1.4%)	564 (2.9%)	1455 (7.5%)	2,347 (3.7%)
Epilepsy	48 (2.9%)	628 (2.7%)	618 (3.2%)	844 (4.4%)	2,138 (3.4%)
Hypertension	40 (2.4%)	767 (3.3%)	1,418 (7.2%)	2,372 (12.3%)	4,597 (7.2%)
Osteoarthritis	21 (1.3%)	419 (1.8%)	630 (3.2%)	1,056 (5.5%)	2,126 (3.3%)
Parkinson	2 (0.1%)	38 (0.2%)	40 (0.2%)	33 (0.2%)	113 (0.2%)

*BMI, Body mass Index*.

The mean age of participants was 40.9 years (*SD* = 15.6 years); the youngest group was underweight patients (32.9 ± 17.2 years) and the oldest were obese (42.1 ± 14.5 years) and overweight (43.3 ± 15.5 years) patients. The mean age for normal weight group was 38.3 ± 15.9 (years). The majority of the sample (70.7%; (95% CI [70.3, 71.0])) were women; the proportion of women vs. men dominated in each weight category (Table 1).

The mean BMI for the sample was 27.8 (*SD* = 6.9) kg/m^2^, with 36.1 (*SD* = 6.2) kg/m^2^ in obese patients and 22.2 (*SD* = 1.7) kg/m^2^ in normal weight patients. Obese patients had a substantially higher prevalence of comorbidities (20.2%; (95% CI [19.6, 20.7])) than normal weight patients (8.1%; (95% CI [7.8, 8.5])). For the total sample, the most prevalent comorbidity was hypertension (7.2%; (95% CI [7.0, 7.4])), followed by diabetes (3.7%; (95% CI [3.5, 3.8])), epilepsy (3.4%; (95% CI [3.2, 3.5])) and osteoarthritis (3.3%; (95% CI [3.2, 3.5])). In obese patients, hypertension (12.3%; (95% CI [11.8, 12.8])) and diabetes (7.5%; (95% CI [7.2, 7.9])) were substantially more prevalent than for the whole sample.

### Antidepressants Prescribing

**Figure 1:**
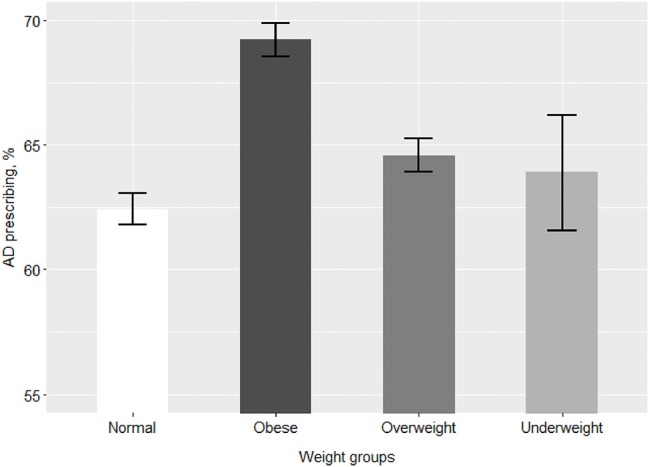
Prevalence of prescribing at least one AD among patients of different weight groups. AD, antidepressant medications. The bars represent % prevalence and 95% confidence intervals of prescribing at least one AD for patients of each weight group.

Of the 63,830 patients with depression, 41,606 were prescribed at least one AD during the study period. Table 2 and Figure 1 describe the period prevalence of prescribing at least one AD within 2011–2016 for patients of different weight categories diagnosed with depression. The prevalence of AD prescribing was higher among obese patients and overweight patients than among normal weight patients (Table 2). There was no difference in prescribing for underweight patients.

**Table 2:** Prevalence of prescribing at least one AD to patients with depression, according to the weight category and sex.

**Weight group**	**Number of patients in the group**	**Prevalence of AD prescribing for each weight group**		
		**Number of patients with AD prescriptions**	**% prevalence**	**95% CI**
Underweight	1,685	1,077	63.9	61.6, 66.2
Men	380	238	62.6	57.5, 67.5
Women	1,305	839	64.3	61.6, 66.9
Normal	23,188	14,476	62.4	61.8, 63.1
Men	5,569	3,535	63.5	62.2, 64.7
Women	17,619	10,941	62.1	61.4, 62.8
Obese	19,314	13,369	69.2	68.6, 69.9
Men	5,791	4,078	70.4	69.2, 71.6
Women	13,523	9,291	68.7	67.9, 69.4
Overweight	19,643	12,684	64.6	63.9, 65.2
Men	6,982	4,522	64.8	63.6, 65.9
Women	12,661	8,162	64.5	63,6, 65.3
Total	63,830	41,606	65.2	64.8, 65.6
Men	18,722	12,373	66.1	65.4, 66.8
Women	45,108	29,233	64.8	64.4, 65.3

*AD, antidepressant medications; 95% CI, 95% confidence intervals*.

**Figure 2:**
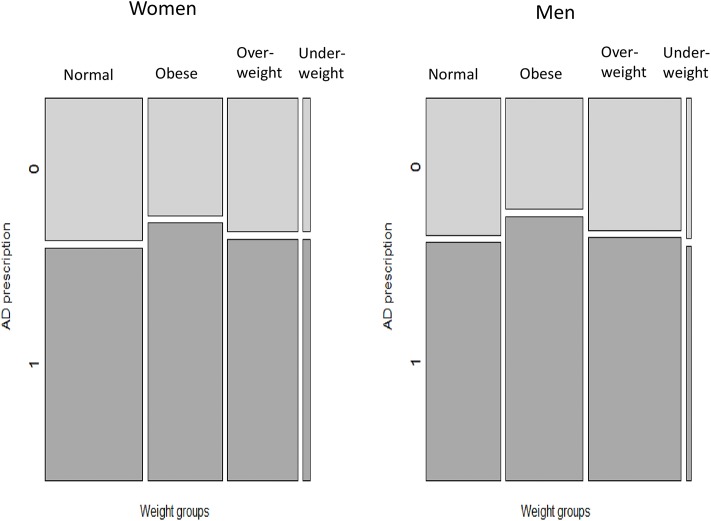
Prevalence of AD prescribing among patients of different weight categories, according to sex. AD, antidepressant medications. The bars (Normal, Obese, Overweight, Underweight) represent weight categories; thickness of the bars represent a proportion of patients in each weight category. Dark tiles represent proportions of patients with AD prescriptions (“1”), light tiles—proportions of patients without AD prescriptions (“0”) in each weight category.

With regard to differences in sex for patients of different weight categories prescribed AD, the proportion of overweight women receiving AD was slightly higher than the proportion of women with normal weight prescribed AD; however, this difference was not clinically meaningful (Table 2). For obese and underweight patients, there was no difference in sex regarding AD prescribing. These patterns are demonstrated by a mosaic plot (Figure 2). The plot also shows that the distribution of weight categories (thickness of the bars) is different for men and women: the prevalence of normal weight patients is higher in women and the prevalence of overweight patients is higher in men.

**Figure 3:**
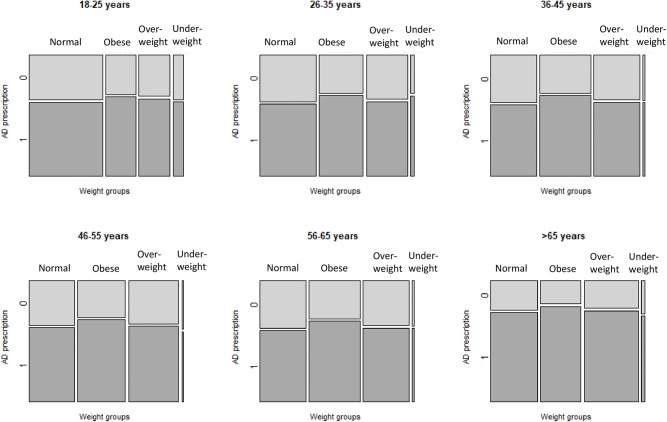
Prevalence of AD prescribing, according to weight category and age group. AD, antidepressant medications. Each mosaic plot represents an age group. The bars (Normal, Obese, Overweight, Underweight) represent weight categories; thickness of the bars represent a proportion of patients in each weight category. Dark tiles represent proportions of patients with AD prescriptions (“1”), light tiles—proportions of patients without AD prescriptions (“0”) in each weight category.

Supplementary Table 2 shows sociodemographic and clinical characteristics for patients with AD prescriptions belonging to different weight categories. The lowest mean value for age was for the category of the underweight patients. The mean age of normal weight patients prescribed AD was 39.0 ± 16.5 (years), and the mean age of obese patients with AD prescriptions was 42.8 ± 14.8 (years). In all weight categories, mean age of those without AD prescriptions was lower than patients with prescriptions (data not shown). Supplementary Table 2 and Figure 3 illustrate differences in AD prescribing for patients of different weight categories and age groups. For all age groups, the proportion of obese patients with AD prescription was higher than the proportion of patients without AD prescriptions. This difference, however, is subtler for seniors (patients >65 years of age).

Patients who had at least one comorbidity had a higher prevalence of AD prescribing than patients without any comorbidities for all weight categories. Supplementary Table 2 and Supplementary Figure 1 also show that the proportion of patients with comorbidities (the thickness of the bars) was the highest in obese patients.

### Associations Between Obesity Status and AD Prescribing

Table 3 shows crude and adjusted odds ratios for the association between the weight category and AD prescribing. After adjusting for covariates and clustering by network, obese patients were 17% more likely (95% CI: 1.12, 1.22) and overweight patients were 5% more likely (95% CI: 1.00, 1.09) to receive AD prescriptions compared with normal weight patients. For underweight patients, the results were inconclusive. Seniors (patients >65 years old) were 16% (95% CI: 1.07, 1.27) more likely to receive AD compared to the youngest patients (18–25 years old). Sex was not a significant predictor of prescribing after adjusting for other factors. Including the variable representing rural vs. urban type of residence did not affect the results; therefore, this variable was not retained in the final multivariable model. Receiver operating characteristic (ROC) curve for the model is shown on Supplementary Figure 4A.

**Table 3:** Univariable and multivariable logistic regression analyses of the association between patient's weight category and AD prescribing among CPCSSN patients with depression, adjusted for clustering by networks.

**Variables**	**Weight category**	**Logistic regression,** **unadjusted to network ID**				**Mixed effects model with** adjustment for clustering^**^	
		**cOR**	**95% CI**	aOR^*^	**95% CI**	aOR^*^	**95% CI**
Weight group	Normal weight (Ref)	1	–	1	–	1	–
	Underweight	1.07	0.96, 1.18	1.05	0.95, 1.16	1.02	0.91, 1.13
	Overweight	1.10	1.05, 1.14	1.06	1.02, 1.11	1.05	1.00, 1.09
	Obese	1.35	1.30, 1.41	1.23	1.18, 1.28	1.17	1.12, 1.22
Sex	Women (Ref)	1	–	1	–	1	–
	Men	1.06	1.02, 1.10	1.00	0.96, 1.04	0.98	0.95, 1.02
Age (years)	18–25 (Ref)	1	–	1	–	1	–
	25–35	0.99	0.94, 1.04	0.95	0.90, 1.00	1.00	0.95, 1.06
	35–45	0.99	0.94, 1.04	0.91	0.86, 0.96	0.98	0.93, 1.04
	45–55	1.06	1.00, 1.12	0.92	0.87, 0.97	0.98	0.93, 1.03
	55–65	1.18	1.11, 1.26	0.91	0.85, 0.97	0.94	0.88, 1.00
	>65	1.91	1.76, 2.06	1.13	1.04, 1.23	1.16	1.07, 1.27

*Complete cases analysis*.

*AD, antidepressant medications; cOR, crude odds ratio; aOR, adjusted odds ratio*.

* *Adjusted also for the following comorbidities: hypertension, diabetes, epilepsy, osteoarthritis, COPD, Parkinson's disease, and dementia*.

** *Adjusted for clustering by networks*.

After multiple imputation with MICE (Supplementary Table 3) for the total sample of 120,381 patients, there was not significant or substantial change in results. Likewise, there were no substantial or significant changes in the model after adjusting for smoking status (data not shown).

### Subgroup Analysis: AD Prescribing for Obese Patients According to the Obesity Class

Of 19,314 obese patients in our sample, 55.8% (10,782 patients) belonged to obesity class I, 25.2% (4,869) to obesity class II, and 19% (3,663) to obesity class III. There were fewer patients in obesity class I and more patients in higher obesity classes among women than among men (Supplementary Figure 2, thickness of the bars). Supplementary Figure 2 show that higher proportions of men and women in higher obesity classes (II and III) received AD than patients in obesity class I.

Table 4 shows crude and adjusted odds ratios for the association between obesity class and AD prescribing, with and without adjustment for network clustering. After adjusting for comorbidities and clustering by networks, patients with obesity classes II were 8% more likely (95% CI: 1.00, 1.16) and patients with obesity class III were 6% (95% CI: 0.98, 1.16) more likely to receive AD. Neither sex nor age were important factors in the association between prescribing and obesity class after adjusting for other factors and clustering by networks. ROC curve for the model is shown on Supplementary Figure 4B.

**Table 4:** Univariable and multivariable regression analyses of the association between obesity class and AD prescribing among CPCSSN patients with depression and obesity.

**Variables**	**Obesity class**	**Logistic regression,** **unadjusted to network ID**				Mixed effects model with adjustment for clustering^**^	
		**cOR**	**95% CI**	aOR^*^	**95% CI**	aOR^*^	**95% CI**
Weight group	Class I (Ref)	1	–	1	–	–	–
	Class II	1.13	1.05, 1.22	1.10	1.02, 1.19	1.08	1.00, 1.16
	Class III	1.17	1.08, 1.28	1.10	1.01, 1.19	1.06	0.98, 1.16
Sex	Women (Ref)	1	–	1	–	–	–
	Men	1.08	1.01, 1.16	1.03	0.96, 1.10	1.01	0.95, 1.09
Age (years)	18–25 (Ref)	1	–	1	–	1	–
	25–35	1.06	0.95, 1.18	1.03	0.93, 1.14	1.07	0.96, 1.20
	35–45	1.06	0.96, 1.18	0.99	0.89, 1.10	1.05	0.95, 1.17
	45–55	1.13	1.01, 1.25	0.95	0.85, 1.06	1.00	0.89, 1.12
	55–65	1.33	1.18, 1.55	0.94	0.83, 1.07	0.97	0.85, 1.11
	>65	2.04	1.74, 2.40	1.09	0.92, 1.30	1.14	0.95, 1.36

*Complete cases analyses*.

*AD, antidepressant medications; cOR, crude odds ratio; aOR, adjusted odds ratio*.

* *Also adjusted for comorbidities*.

** *Adjustment for clustering with networks as clusters*.

As compared to the CC analysis, after multiple imputation with MICE, there were no changes in neither effect estimate nor 95% CI for obesity class II, and there were non-substantial and non-significant changes for obesity class III (Supplementary Table 4). No significant or substantial changes after adjusting for smoking status were observed (data not shown).

### Prescribing by PCP From Different Networks

When the analysis was stratified by networks, all networks showed increased odds for patients with obesity, compared to normal weight patients, to receive AD prescriptions, with the exception of one network (Table 5, network E) for which the results were inconclusive.

**Table 5:** Multivariable logistic regression analyses of the association between patient's weight category and AD prescribing among CPCSSN patients with depression, according to networks.

**Network ID**	***A***	***B***	***C***	***D***	***E***	***F***	***G***	***H***	***I***	***J***	***K***	***L***
aOR^*^; 95% CI	1.08; 0.99, 1.18	1.14; 1.00, 1.30	1.14; 0.99, 1.31	1.21; 1.06, 1.38	0.69; 0.23, 2.03	1.33; 0.86, 2.05	1.15; 1.05, 1.27	1.31; 1.06, 1.63	1.26; 1.02, 1.55	1.64; 1.13, 2.38	1.26; 1.01, 1.53	1.42; 1.08, 1.86

*AD, antidepressant medications; aOR, adjusted odds ratio; 95% CI, 95% confidence intervals*.

* *Adjusted to age, sex, and all comorbidities*.

### The Number of Different AD Types Prescribed

**Figure 4:**
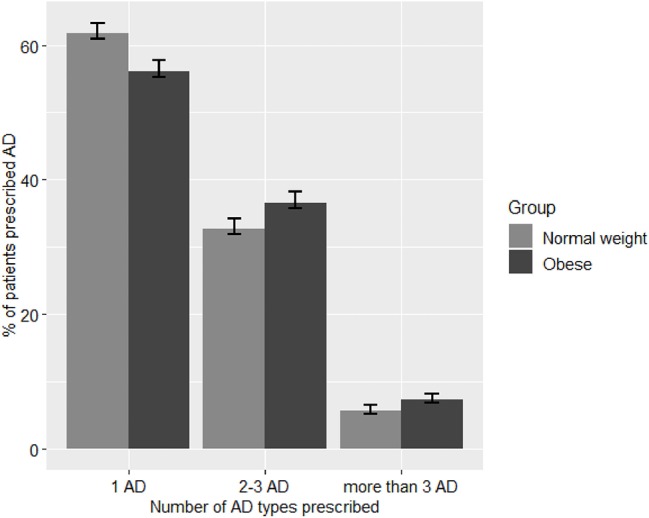
Number of different AD types prescribed to patients with obesity and normal weight patients. AD, antidepressants medications. The bars represent % prevalence and 95% confidence intervals of prescribing 1, 2, or >3 different types of AD for either obese or normal weight categories.

Figure 4 shows the difference in prevalence of prescribing either 1, 2–3, or >3 AD types between patients with different weight categories with AD prescriptions. Compared with normal weight patients, the prevalence of prescribing >3 AD was higher in the obese category (7.3% (95% CI [6.8, 7.7]) than in the normal weight category (5.6% (95% CI [5.2, 6.0])). Likewise, the prevalence of prescribing 2-3 AD was higher in the obese category (36.6% (95% CI [35.8, 37.5]) than in the normal weight category (32.7% (95% CI [31.9, 33.5]). Conversely, prevalence of prescribing only one AD was lower in obese patients (56.1% (95% CI [55.3, 56.9]) than in normal weight patients (61.7% (95% CI [60.9, 62.5]). This pattern seems to be more prominent in women than in men as demonstrated by the mosaic plots on Supplementary Figure 3.

**Figure 5:**
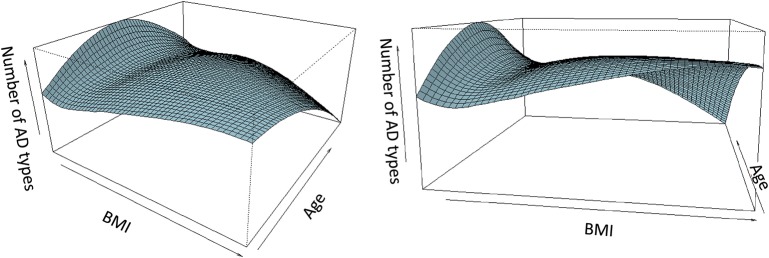
Number of different AD types prescribed to a patient, in relation to BMI and age. AD, antidepressant medications; BMI, body mass index. Two different views (at different angles) of the same smooth surface plot are presented. The plot represents relationships between the number of different AD types prescribed to a patient, patient's BMI, and patient's age. Each horizontal line represents an age group. For young patients, the number of different AD increases with increasing BMI. For middle aged patients, the relationship is close to U-shaped. For old patients, the number of AD types prescribed decreases with an increase in BMI.

The smooth surface plot on Figure 5 shows how the number of AD types prescribed changes between different BMIs in relation to age. Each horizontal line on the plot corresponds to an age group. As the figure shows, the number of different AD types increases with an increase in BMI for young patients. For middle aged patients, this relationship is less prominent. For old patients with a very high BMI, the number of AD types prescribed to a patient decreases.

## Discussion

In our study, we evaluated the prevalence of AD prescribing among primary care patients with depression in Canada belonging to different weight categories in eight Canadian provinces and one territory, and we examined the association of prescribing prevalence with obesity status and with obesity class. We observed that primary care patients with obesity were more likely to receive pharmacological treatment for depression than normal weight patients, with the highest odds for morbidly obese patients (classes II and III), and that a greater proportion of obese patients received prescriptions for a high number (more than three) of different AD types than did normal weight patients. These relationships are not modified by sex but may depend on patient's age.

Our results are in line with the studies conducted in the UK and the USA (32, 33) which also reported high prevalence of AD prescribing in obese patients with depression. One of our main findings was that, after adjusting for covariates and clustering by networks, people with depression and comorbid obesity were 17% (95% CI [1.12,1.22]) more likely than normal weight patients to receive pharmacological treatment with AD. Despite differences between the networks, possibly related to the socio-demographic characteristics and beliefs of patients and doctors between provinces, as well as to the differences in drug insurance coverage and access to medical help, nationwide in Canada, obese patients with depression were more likely to be prescribed pharmacological treatment using AD. For only one network, the results were inconclusive, probably due to the small number of patients.

It is still unclear whether these findings reflect more severe form of depression in patients with obesity that requires pharmacological treatment, or the attitudes and beliefs of PCP that lead them to prescribe pharmacological treatment to people with obesity more often than to normal weight patients. In support of the former, obesity was associated with more severe depression (34–36), especially in extremely obese patients (37). Patients with obesity may need dose adjustment and a longer treatment duration to reach the same level of response as non-obese patients (38–41). Since our population is a population of prevalent users and our analysis is cross-sectional, longer treatment duration for obese patients may have contributed to the prevalence of both obesity and AD prescribing in this group.

It is possible, however, that obesity bias contributes to this pattern of treatment: obese patients may be considered by some PCP as unmotivated and non-adherent to recommendations for behavioral changes (42) and, therefore, less likely to respond to psychotherapy. Therefore, they prescribe medications. In support of this hypothesis, it has been previously reported that obese patients in the USA were less likely to receive psychotherapy as a treatment for a new depression episode (21), possibly due to the health providers and/or patients' bias on the efficacy of counseling in obese patients (21). It is known that negative attitudes toward obese patients can influence decision-making by medical professionals and impact the care they provide (19, 43). Rejection of certain treatments for obese patients in different countries worldwide has become a problem highlighted by several studies (44, 45). Moreover, it has been recently shown in a qualitative study (46) that health professionals who had weight bias “used less teaching discourse” for obese patients and started them on pharmaceutical therapies sooner. Qualitative studies are needed to find out whether health professionals often go straight to prescribing pharmacological therapy to obese patients, bypassing the psychotherapy option. Such behavior is particularly important to combat because of the obesogenic properties of AD which can increase the risk for patients with class I to be “promoted” to higher obesity classes.

Of importance, patients with high obesity classes are more likely to have multiple comorbidities (47) and higher mortality rates (48). In addition, they are more likely to suffer from the obesity stigma leading to low self-esteem (49) and are at increased risk for depressed mood (50–52). In our study, patients with morbid obesity had higher odds of receiving AD. This may be attributed to their elevated risk for more severe depression that needed pharmacological treatment. On the other hand, it can be attributed to the higher prevalence of obesity bias toward morbidly obese patients (5, 53).

These reasonings, however, should be considered with caution: the cross-sectional nature of our study (our study design was limited by the database restrictions) does not allow us to account for the temporality of findings. We cannot state with certainty whether AD were initially prescribed to obese patients or if prescribing AD contributed to a greater proportion of obese patients with AD prescriptions. The latter possibility, however, is of equal concern, since utilization of AD may contribute to increasing the risk of a long-term weight gain at the population level, moving normal weight and overweight patients to the obesity group (22). It has been reported that at least 1.5% of the obesity rate increase among young adults in the USA during the last two decades can be explained by the increase in the prevalence of depression and AD use (54). Of note, even though the receiver operating (ROC) curves did only show moderate predictive capability for our model (Supplementary Figure 4), our purpose was not to predict prescribing. The statistical models were employed to establish direction and magnitudes of associations between obesity (and other important patient's characteristics), and prescribing. The relatively low model prediction accuracy indicates that other important predictors (e.g., type and severity of depression, physicians' preferences etc.) need to be included for better predictive capacity, this will require further research.

Another important finding was an increased prevalence of prescribing a high number of different AD types by PCP to obese patients compared with normal weight patients. It is possible that patients with obesity have more complex disease with a number of specific features that require concurrent prescribing of more than one AD. It may also be explained by a greater prevalence of treatment resistant depression in this population (4) requiring a high number of switches from one AD to another. Resistance to treatment with AD in obese patients with depression may be caused by an interplay of multiple factors. One of them may be the reduced bioavailability of AD, the drugs with a relatively high lipophilicity, due to excess of adipose tissue in obese patients (55, 56). This may lead to lower plasma concentrations and, potentially, an attenuated therapeutic effect. In addition, contributing roles of inflammatory cytokines (53, 57–60) and adipokines (61–63), as well as genetic factors (64–67) were proposed. These players may have an impact on drug metabolism and dysregulation of hypothalamic pituitary axes and cell signaling pathways which modifies the response to therapy. Different response to certain groups and types of AD in patients with excess weight, as compared to normal weight patients, was reported in several studies and described in two recent reviews (6, 41). Our findings, therefore, may reflect physicians' difficulties with selection of an effective AD medication for obese patients with depression.

Of note, the relationships between the number of different AD types prescribed and patient's BMI may depend on age, as illustrated by the smooth surface plot on Figure 5. For very young patients, in general, the number of prescribed AD types increases with the increase of BMI. This may reflect particular difficulties with a choice of AD to treat depression requiring a high number of switches in people with excess weight at a young age. This observation deserves further evaluation since certain types of AD were shown to be associated with the increased risk of suicides in this particular age group (68–70); therefore, choosing the most effective AD without a high number of switches may help decrease this risk. For middle aged patients, the relationship between AD number and BMI is less prominent. Moreover, for this age group, the relationship is close to U-shaped, with higher number of AD types prescribed to people with a very low and a very high BMI. This observation may reflect difficulties with the choice of appropriate medication not only in the obese but also in the underweight group that warrants corresponding investigation. Contrary to the youngest group, for older participants, the number of prescribed AD types decreases with the increase of BMI. Old patients with obesity may have higher number of comorbidities and receive higher number of different medications, compared with their younger counterparts. Therefore, PCP may try to avoid concurrent prescribing of more than one AD type to the elderly to decrease the probability of side effects of drug-drug interactions due to polypharmacy, which is in line with the guidelines on AD prescribing in older population (8). Of note, patients of the oldest group (>65 years old) were more likely to receive at least one AD prescription, compared with the youngest group of patients, even when the odds ratio for age was adjusted for the obesity status. Considering that our sample includes prevalent users, these results, at least in part, can be related to the fact that older patients are more likely to have relapses and may be less likely to reach an adequate response to treatment than their young counterparts (8).

Our results suggest that obesity may be one of the important factors that require an individualized approach to pharmacological treatment of depression in all age groups. Recent evidence on different responses to certain AD in obese patients compared with normal weight patients (5, 6, 38, 39, 53, 71, 72) cannot be disregarded. Currently, there are no guidelines but there are several recent studies and reviews that contain clinically relevant information on the difference in response to certain AD in patients with obesity and with certain obesity classes (5, 53, 72, 73). There are also published detailed recommendations on how to avoid the weight-increasing effect of AD (11, 22, 54, 74–80). Of note, it has been shown in a recent RCT that patients with morbid obesity may benefit from certain AD and AD combinations (5, 53) and, therefore, require individualized approach to treatment. These recommendations, however, are not included in the guidelines, and, therefore, may be unknown to a wide community of primary care physicians. All this evidence needs to be synthetized and appraised so that experts can consider whether its quality and strength allows the addition of obesity-specific recommendations to the guidelines. Our results show that the risk of adverse effects due to drug-drug interactions in obese patients at increased risk for polypharmacy seems to be accounted for only when prescribing different AD types for older patients but not in the young or the middle-aged group. Better guidelines on the individualized selection of AD for patients with depression and comorbid obesity would help optimize AD treatment in obese patients with depression and may help decrease the number of adverse effects due to drug-drug interactions.

Our study has certain strength and limitations. First, the CPCSSN depression detection algorithm detects lifetime depression, precluding one from distinguishing between prevalent or incident cases. In line with this limitation, our study has a cross-sectional design, and we discussed our findings in the light of the limitations of a cross-sectional study. Second, the information on socio-economic status (SES) is not recorded in the CPCSSN database; therefore, we were not able to adjust our models for it. We, however, were able to adjust for the urban/rural residency as a proxy of SES, using postal codes. One of the limitations is that the information on type and severity of depression, as well as a number of important lifestyle variables, such as diet and exercising, are not recorded in the database making it impossible to adjust for these salient variables. Another potential confounder which we could not adjust for due to the lack of reliable information in our database is the diagnosis of an eating disorder in a patient with depression. Certain eating disorders are indications for AD prescribing. Including patients with eating disorders who maintained normal weight in our reference (i.e., normal weight) group could lead to underestimation of the association between obesity and prescribing AD for depression. If a substantial proportion of patients with eating disorders (e.g., bulimia nervosa, binge eating, or night eating) were obese or overweight, this could lead to overestimating the association between excess weight and prescribing AD for depression. Most often, however, people with eating disorders are either underweight or have normal weight (81). In addition, prevalence of eating disorders among adult primary care patients is low in Canada (82), and we do not expect a substantial proportion of patients with this diagnosis in our sample. Therefore, the confounding effect of this variable is not likely to have a substantial impact on our results. In addition, pregnant women or patients with cancer who can experience substantial weight changes, were not excluded as identifying them in the CPCSSN database was not feasible. This could also have confounded our results. Finally, one of the serious limitations of our study was a high proportion of missing data on weight in our database, as well as on smoking status. To deal with this problem, we used the MICE technique to impute missing data and re-evaluated associations between excess weight and AD prescribing, and the obesity classes and AD prescribing, to compare with the CC analysis. This sensitivity analysis showed that the size of effect estimates became slightly smaller for the dataset with the imputed data for weight and did not change substantially after adjusting for the smoking status, but the associations kept the same directions and the level of significance.

## Conclusion

In summary, this was the first study to evaluate differences in prevalence and patterns of prescribing AD between obese and normal weight patients, and between patients with different classes of obesity in Canadian primary care. We also describe the association between AD prescribing and obesity, using a large national primary care dataset. It is also the first study to demonstrate consistency in the direction of this association between different networks participating in CPCSSN, showing uniformity of the association across Canadian provinces. In terms of methodology, this was, to our knowledge, the first study where the MICE technique was applied to deal with the substantial proportion of missing data on important clinical variables, such as weight and smoking status, in the national CPCSSN database. Higher prevalence of AD prescribing and prescribing high number of AD to obese patients compared with normal weight patients in all provinces of Canada raises a public health concern. Longitudinal studies are required to evaluate how AD prescribing patterns, including prescribing individual AD groups and types, can be related to obese patient's general health and subsequent heath care utilization. Focus should be on the AD types known for their risk of weight gain and the types that were shown non-effective or less effective for patients with obesity in recent publications. Stakeholders and experts may want to revise the evidence to add recommendations on a different approach to AD selection for patients with obesity, especially for patients with obesity II and III classes. To obtain stronger evidence, more studies should be conducted to evaluate the response to individual AD drugs in obese patients.

## Data Availability Statement

The datasets generated for this study are available on request to the corresponding author.

## Ethics Statement

The studies involving human participants were reviewed and approved by The Institutional Review Board of McGill University. Written informed consent for participation was not required for this study in accordance with the national legislation and the institutional requirements.

## Author Contributions

SP was primary investigator and conducted the research under the supervision of TS and GB. All authors contributed on the methods and interpretation of results. The text was written by SP and revised by the other authors.

### Conflict of Interest

The authors declare that the research was conducted in the absence of any commercial or financial relationships that could be construed as a potential conflict of interest.

## Abbreviations

BMI: Body mass index
AD: Antidepressants
SSRI: Selective serotonin reuptake inhibitors
SNRI: Serotonin-norepinephrine reuptake inhibitors
TCA: Tricyclic antidepressants
MAOI: Monoamine oxidase inhibitors
PCP: Primary care providers
CPCSSN: Canadian Primary Care Sentinel Surveillance Network
MICE: Multiple imputations by chain equations
MI: Multiple imputations
OR: Odds ratio
95% CI: 95% Confidence interval.
