# Supplementary figures and images for: Evaluating Prevalence and Patterns of Prescribing Medications for Depression for Patients With Obesity Using Large Primary Care Data (Canadian Primary Care Sentinel Surveillance Network)
Source: Front Nutr. 2020 Mar 17;7:24. doi: 10.3389/fnut.2020.00024 (PMC7090027; doi:10.3389/fnut.2020.00024)

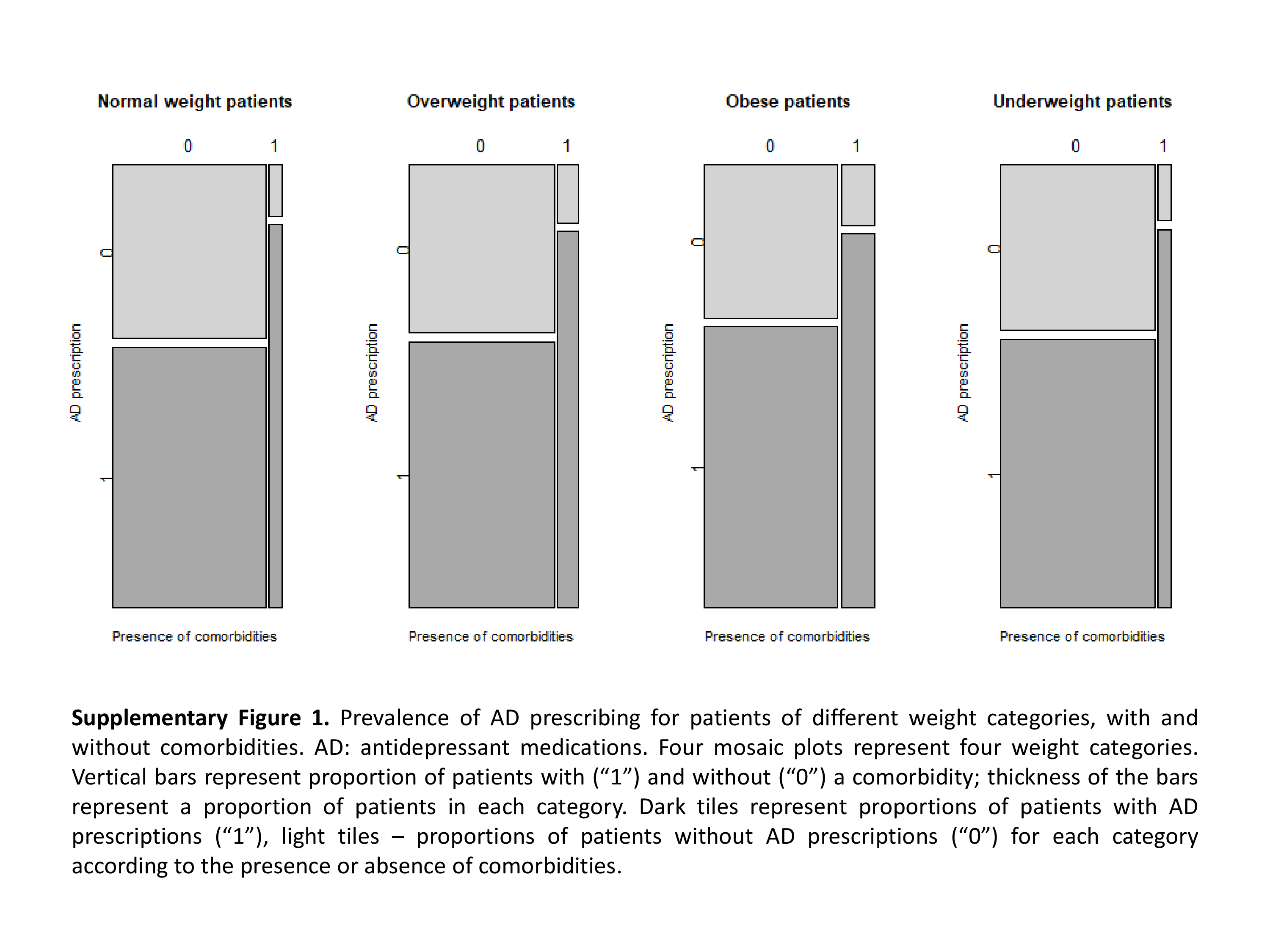

Supplement: Supplementary file 1 [file Image_1.tiff]

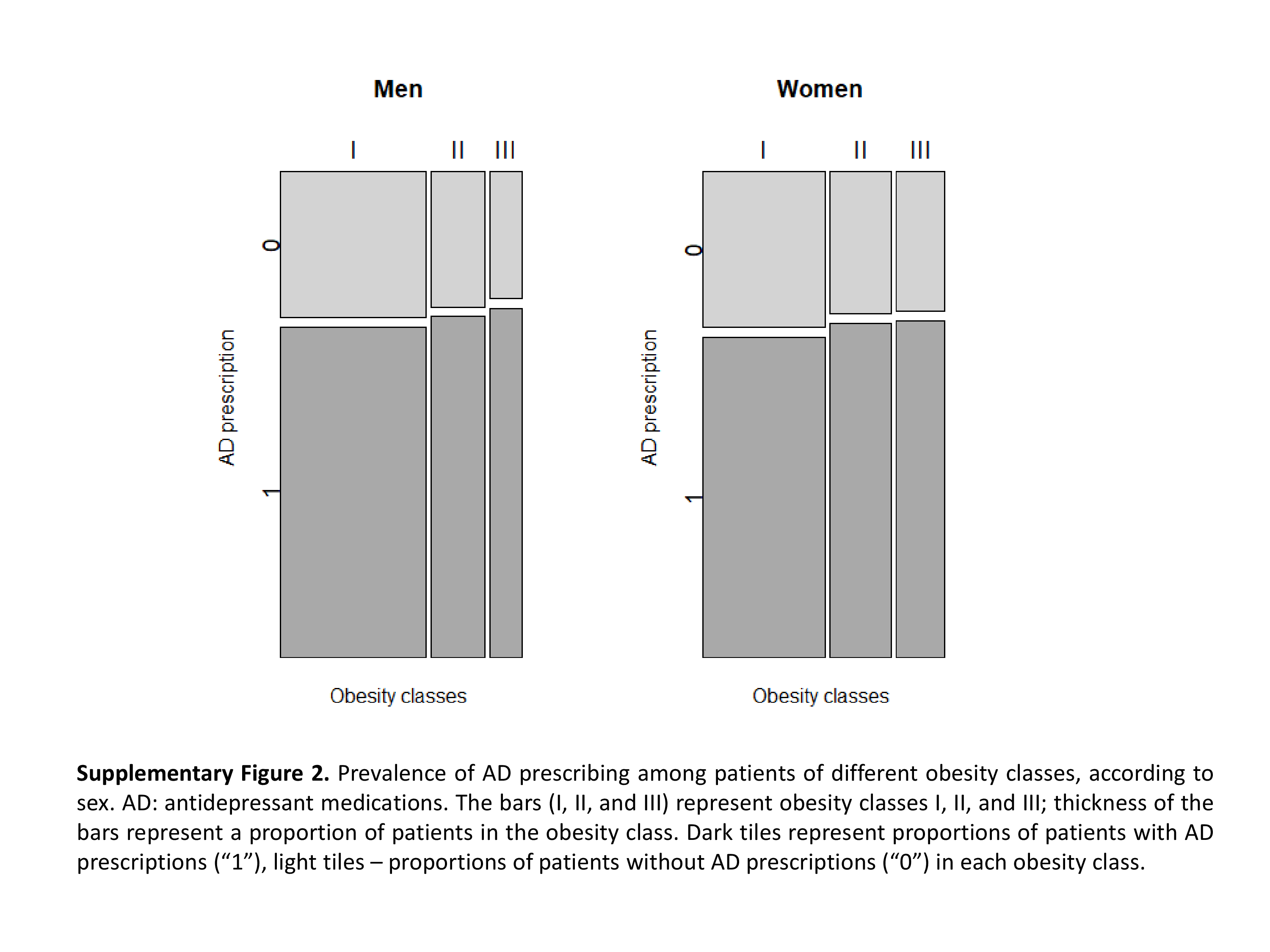

Supplement: Supplementary file 2 [file Image_2.tiff]

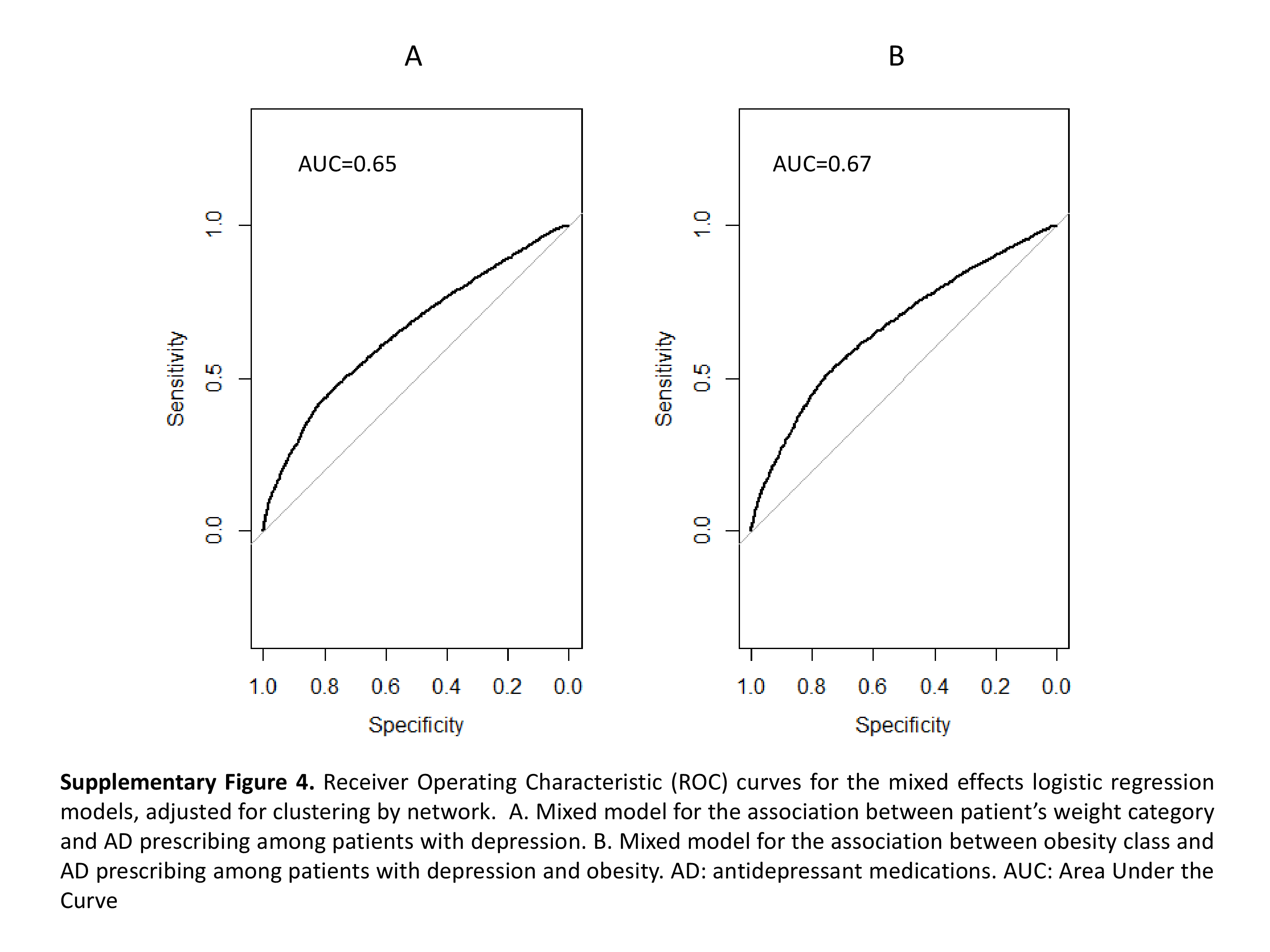

Supplement: Supplementary file 3 [file Image_4.tiff]

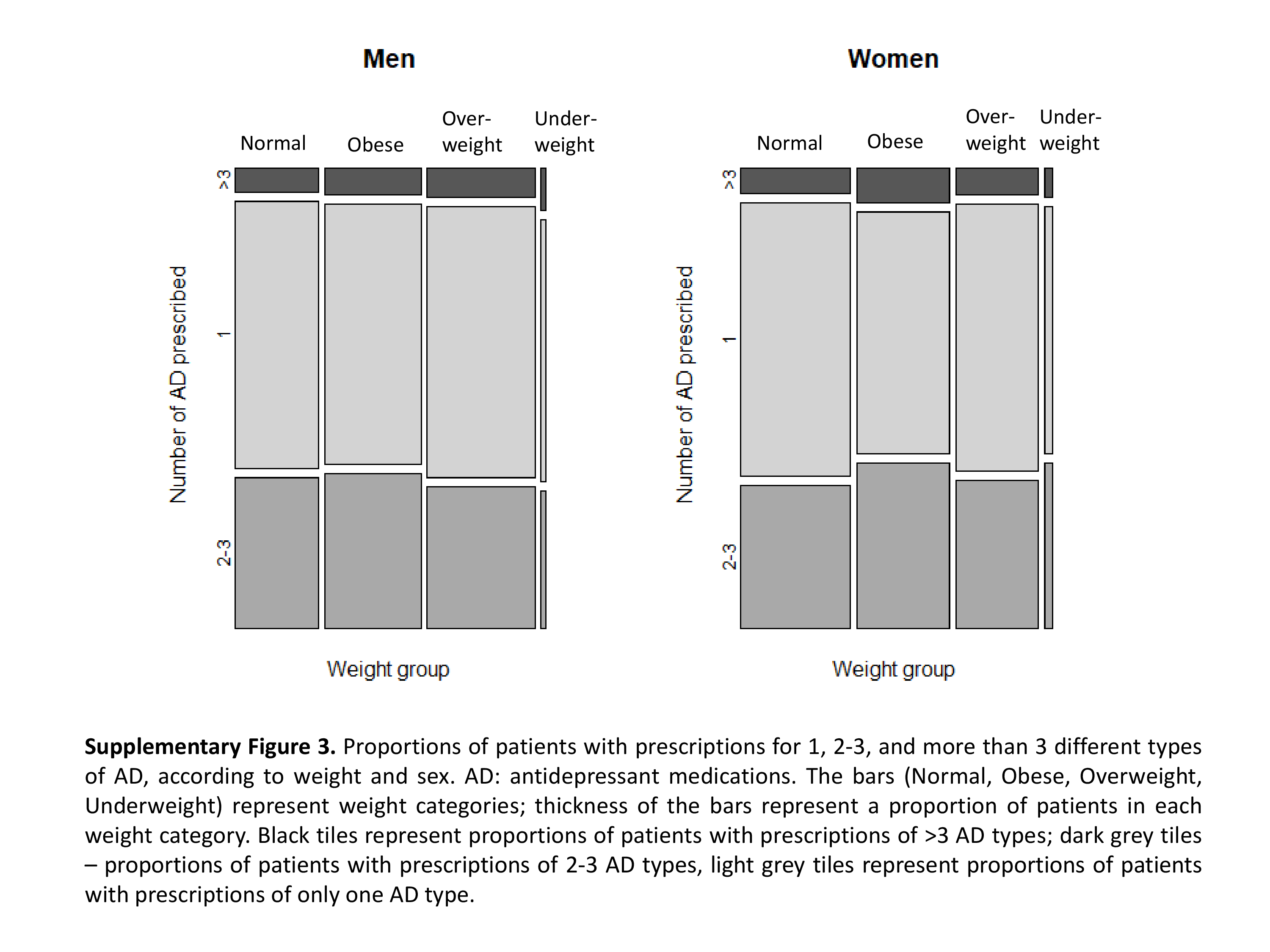

Supplement: Supplementary file 8 [file Figure_3.tiff]
