# Supplementary material for: Evaluating Prevalence and Patterns of Prescribing Medications for Depression for Patients With Obesity Using Large Primary Care Data (Canadian Primary Care Sentinel Surveillance Network)
Source: Front Nutr. 2020 Mar 17;7:24. doi: 10.3389/fnut.2020.00024 (PMC7090027; doi:10.3389/fnut.2020.00024)
Supplement: Supplementary file 4 [file Table_1.pdf]

Supplementary Table 1. Antidepressant medications included in the analysis.

Medications for depression	
Class	Type
Selective Serotonin Reuptake Inhibitors (SSRI)	Escitalopram, citalopram, sertraline, fluoxetine, paroxetine, fluvoxamine
Serotonin-norepinephrine reuptake inhibitors (SNRI)	Venlafaxine, duloxetine, desvenlafaxine
Tricyclic antidepressants (TCA)	Amitriptyline, nortriptyline, doxepin, imipramine, clomipramine, desipramine, trimipramine, amoxapine,
Tetracyclic antidepressant	Maprotiline
Norepinephrine and specific serotonergic antidepressants (NaSSA)	Mirtazapine
Norepinephrine-dopamine reuptake inhibitors (NDRI)	Bupropion
Serotonin antagonist reuptake inhibitors (SARI)	Trazodone, nefazodone, vilazodone, vortioxetine
Second-generation “atypical” antipsychotic	Quetiapine
Irreversible Monoamine oxidase (MAO) inhibitors	Phenelzine, tranylcypromine
Reversible inhibitor of MAO-A	Moclobemide
