# Supplementary material for: Evaluating Prevalence and Patterns of Prescribing Medications for Depression for Patients With Obesity Using Large Primary Care Data (Canadian Primary Care Sentinel Surveillance Network)
Source: Front Nutr. 2020 Mar 17;7:24. doi: 10.3389/fnut.2020.00024 (PMC7090027; doi:10.3389/fnut.2020.00024)
Supplement: Supplementary file 5 [file Table_2.pdf]

Supplementary Table 2. Characteristics of patients prescribed AD, according to weight categories

	Weight category				
	Underweight, N=1,685 (2.6%)	Normal weight N=23,188 (36.3%)	Overweight N=19,643 (30.8%)	Obese N=19,314 (30.3%)	Total N=63,830 n (%)
	AD+ N=1,077 (63.9%) n (%)	AD+ N=14,476 (62.4%) n (%)	AD+ N=12,684 (64.6%) n (%)	AD+ N=13,369 (69.2%) n (%)	AD+ N=41,606 (65,2%) n (%)
Age					
mean (SD)	33.4 (17.5)	39.0(16.5)	44.0 (16.0)	42.8 (14.8)	41.6(16.0)
median (IQR)	26.0 (22.1)	35.7 (25.4)	43.0 (23.5)	41.6 (21.6)	39.9(24.2)
Gender					
Men	238 (22.1)	3535 (24.4)	4,522 (35.7)	4,078 (30.5)	12,373(29.7)
Women	839 (77.9)	10,941 (75.6)	8,162 (64.4)	9,291 (69.5)	29,233(70.3)
BMI, first measure					
mean (SD)	17.4 (0.8)	22.2 (1.7)	27.3 (1.4)	36.2 (6.2)	28.1(7.1)
median (IQR)	17.6 (1.2)	22.4 (2.8)	27.2 (2.5)	34.3 (6.7)	26.8(8.4)
Comorbidities					
At least one comorbidity	110(10.2)	1,665(11.5)	2,129(16.8%)	3,353(25.08)	7257(17.4)
COPD	31 (2.9)	306 (2.1)	279 (2.2)	407 (3.0)	1023(2.5)
Dementia	17 (1.6)	177 (1.2)	214 (1.7)	188 (1.4)	596(1.4)
Diabetes	14 (1.3)	273 (1.9)	496 (3.9)	1247 (9.3)	2030(4.9)
Epilepsy	45 (4.2)	582 (4.0)	577 (4.6)	769 (5.8)	1973(4.7)
Hypertension	34 (3.2)	672 (4.6)	1,220 (9.6)	2,041 (15.3)	3967(9.5)
Osteoarthritis	17 (1.6)	372 (2.6)	538 (424)	903 (6.8)	1830(4.4)
Parkinson	2 (0.2)	35 (0.2)	37 (0.3)	29 (0.2)	103(0.25)3

AD: antidepressant medications; BMI: Body mass Index
