# Supplementary material for: Evaluating Prevalence and Patterns of Prescribing Medications for Depression for Patients With Obesity Using Large Primary Care Data (Canadian Primary Care Sentinel Surveillance Network)
Source: Front Nutr. 2020 Mar 17;7:24. doi: 10.3389/fnut.2020.00024 (PMC7090027; doi:10.3389/fnut.2020.00024)
Supplement: Supplementary file 6 [file Table_3.pdf]

Table 3. Univariable and multivariable logistic regression analyses of the association between patient's weight category and AD prescribing among CPCSSN patients with depression, adjusted for clustering with Networks as clusters. MICE analysis

Variables	Weight Category	Logistic regression, unadjusted to network ID				Mixed effects model with adjustment for clustering**	
		cOR	95% CI	aOR*	95% CI	aOR*	95% CI
Weight group	Normal weight (Ref)	1	-	1	-	1	-
	Obese	1.20	1.14, 1.26	1.14	1.08, 1.21	1.13	1.07, 1.19
	Overweight	1.07	1.03, 1.11	1.04	1.00, 1.08	1.03	0.99, 1.08
	Underweight	1.04	0.93, 1.15	1.05	0.94, 1.16	1.03	0.93, 1.15
Sex	Women (Ref)	1	-	1	-	1	-
	Men	1.02	0.99, 1.04	0.98	0.95, 1.00	0.98	0.95, 1.00
Age (years)	18-25 (Ref)	1	-	1	-	1	-
	25-35	1.07	1.03, 1.11	1.04	1.00, 1.08	1.06	1.02, 1.10
	35-45	1.09	1.05, 1.13	1.02	0.98, 1.06	1.05	1.01, 1.09
	45-55	1.14	1.10, 1.19	1.02	0.98, 1.07	1.04	1.00, 1.08
	55-65	1.24	1.18, 1.30	0.98	0.95,1.05	1.01	0.96, 1.06
	>65	1.71	1.63, 1.81	1.11	1.05, 1.18	1.09	1.03, 1.16

AD: antidepressant medications; cOR: crude odds ratio; aOR: adjusted odds ratio; 95% CI: 95% confidence intervals; MICE: multiple imputations by chained equations

*Adjusted also for the following comorbidities: hypertension, diabetes, epilepsy, osteoarthritis, COPD, Parkinson disease, and dementia; **adjusted for clustering by networks
