# Supplementary material for: Evaluating Prevalence and Patterns of Prescribing Medications for Depression for Patients With Obesity Using Large Primary Care Data (Canadian Primary Care Sentinel Surveillance Network)
Source: Front Nutr. 2020 Mar 17;7:24. doi: 10.3389/fnut.2020.00024 (PMC7090027; doi:10.3389/fnut.2020.00024)
Supplement: Supplementary file 7 [file Table_4.pdf]

Supplementary Table 4. Univariable and multivariable regression analyses of the association between obesity class and prescribing of AD
among CPCSSN patients with depression and obesity. MICE analysis

Variables	Obesity class	Logistic regression, unadjusted to network ID				Mixed effects model with adjustment for clustering**	
		cOR	95%CI	aOR*	95%CI	aOR*	95%CI
Weight group	Class I (Ref)	1	-	1	-	-	-
	Class II	1.12	1.04, 1.19	1.09	1.02, 1.17	1.08	1.01, 1.16
	Class III	1.11	1.02, 1.21	1.05	0.95, 1.16	1.03	0.93, 1.13
Sex	Women (Ref)	1	-	1	-	-	-
	Men	1.01	0.95, 1.07	0.98	0.92, 1.03	0.98	0.93, 1.03
Age (years)	18-25 (Ref)	1	-	1	-	1	-
	25-35	1.13	1.00, 1.27	1.10	0.98, 1.23	1.11	0.99, 1.24
	35-45	1.12	1.00, 1.26	1.06	0.94, 1.19	1.08	0.96, 1.22
	45-55	1.19	1.07, 1.32	1.04	0.93, 1.16	1.05	0.94, 1.17
	55-65	1.32	1.16, 1.49	1.02	0.94, 1.22	1.02	0.89, 1.17
	>65	1.71	1.52, 1.93	1.07	0.92, 1.04	1.05	0.92, 1.19

AD: antidepressants; cOR: crude odds ratio; aOR: adjusted odds ratio; 95% CI: 95% confidence intervals; MICE: multiple imputations by chained equations

*Also adjusted to comorbidities; **adjustment for clustering by networks
